# Changes in the micromorphology of the corneal subbasal nerve plexus in patients after plaque brachytherapy

**DOI:** 10.1186/1748-717X-8-136

**Published:** 2013-06-07

**Authors:** Andrey Zhivov, Karsten Winter, Sabine Peschel, Oliver Stachs, Andreas Wree, Guido Hildebrandt, Rudolf Guthoff

**Affiliations:** 1 Department of Ophthalmology, University of Rostock, D-18055, Rostock, Germany; 2 Translational Centre of Regenerative Medicine, University of Leipzig, Leipzig, Germany; 3 Department of Anatomy, University of Rostock, Rostock, Germany; 4 Department of Radiotherapy and Radiation Oncology, University of Rostock, Rostock, Germany

**Keywords:** Confocal microscopy, Subbasal nerve plexus, Cornea, Brachytherapy, Radiation neuropathy

## Abstract

**Background:**

To quantify the development of radiation neuropathy in corneal subbasal nerve plexus (SNP) after plaque brachytherapy, and the subsequent regeneration of SNP micromorphology and corneal sensation.

**Methods:**

Nine eyes of 9 melanoma patients (ciliary body: 3, iris: 2, conjunctiva: 4) underwent brachytherapy (ruthenium-106 plaque, dose to tumour base: 523 ± 231 Gy). SNP micromorphology was assessed by in-vivo confocal microscopy. Using software developed in–house, pre-irradiation findings were compared with those obtained after 3 days, 1, 4 and 7 months, and related to radiation dose and corneal sensation.

**Results:**

After 3 days nerve fibres were absent from the applicator zone and central cornea, and corneal sensation was abolished. The earliest regenerating fibres were seen at the one-month follow-up. By 4 months SNP structures had increased to one-third of pre-treatment status (based on nerve fibre density and nerve fibre count), and corneal sensation had returned to approximately two-thirds of pre-irradiation values. Regeneration of SNP and corneal sensation was nearly complete 7 months after plaque brachytherapy.

**Conclusions:**

The evaluation of SNP micromorphology and corneal sensation is a reliable and clinically useful method for assessing neuropathy after plaque brachytherapy. Radiation-induced neuropathy of corneal nerves develops quickly and is partly reversible within 7 months. The clinical impact of radiation-induced SNP damage is moderate.

## Background

Plaque brachytherapy is an established modality in the treatment of uveal and conjunctival melanoma. While iodine-125 and ruthenium-106 (Ru-106) have each been used for plaque brachytherapy [1-5], the advantages of plaques containing Ru-106 as a beta-emitter include a finite range of radiation [6]. Various ophthalmological complications following Ru-106 irradiation have been reported in series of patients with malignant uveal melanoma [5,7]. Summanen et al. [7] investigated radiation-related complications in 100 eyes, including 38 with anterior melanoma location (at least partly anterior to the equator, with or without ciliary body involvement). At follow-up after 2 years the probability of developing complications was reported as follows: radiation maculopathy 15%, optic neuropathy 10%, vitreous haemorrhage 9%, secondary (neovascular) glaucoma 4%, and radiation cataract 21%, depending on tumour location. We are not aware of any studies that have investigated corneal sensation problems as well as changes in the micromorphology of the corneal subbasal nerve plexus (SNP) as signs of radiation neuropathy.

Confocal laser scanning microscopy permits in-vivo investigation of tissue in the healthy and pathological cornea. The normal structure of the SNP and its physiological dynamic changes over time have already been studied [8-10]. Similarly, postoperative changes in the micromorphology of the SNP have been described after corneal grafting [11-13] and refractive surgery [11,14], and have been correlated with corneal sensation. Recently, the development of small-fibre neuropathy has been investigated using confocal microscopy in diabetic patients [15,16] and in those with Fabry disease [17]. Our group has established 3-dimensional image reconstruction of the cornea, as well as real-time mapping of large-scale 2-dimensional images of the SNP [18]. This methodology permits the quantitative analysis of corneal nerve structures by confocal microscopy [19].

The aim of the present study was to investigate and quantify the degeneration and regeneration of fibres of the corneal SNP after Ru-106 plaque brachytherapy, and to relate these to corneal sensation changes.

## Methods

This prospective case series study was conducted after a positive appraisal by the local ethics Board of Medical Faculty, University of Rostock, Germany. The research adhered to the tenets of the Declaration of Helsinki. The study was explained in detail to the patients, and informed consent was obtained before any investigative procedures were conducted. The inclusion criteria were absence of current or previous local or systemic disease that could affect the cornea, and a negative history of ocular infections, eye surgery or trauma.

The study was conducted in 9 eyes (3 with ciliary body melanoma, 2 with melanoma of the iris, and 4 with malignant conjunctival melanoma) of 9 patients (aged 61.0 ± 12.1 years; 6 female, 3 male). All patients underwent brachytherapy with an Ru-106 ophthalmic plaque (BEBIG GmbH, Berlin, Germany). Demographic data as well as clinical and dosimetric information are presented in Table 1.

**Table 1:** Demographic data, dosimetric information and clinical history

**Patient**	**Gender**	**Age (years)**	**Location of melanoma**	**Size of tumour (max. height in mm)**	**Dose rate (Gy/h) applicator zone**	**Prescribed dose (Gy) to base in applicator zone**	**Calculated dose**		
							**Cornea adjacent to plaque**	**Central zone**	**Distal zone**
1	F	73	conjunctiva	0.5	4.30	278	1.22	0.33	0.04
2	M	65	conjunctiva	7.0	4.64	293	1.29	0.35	0.04
3	F	46	iris	2.0	5.00	550	2.42	0.66	0.08
4	F	50	iris	3.0	5.09	520	2.29	0.62	0.08
5	F	76	ciliary body	3.6	4.94	527	2.31	0.63	0.08
6	F	40	conjunctiva	1.0	7.79	387	1.7	0.46	0.06
7	F	66	ciliary body	5.0	9.54	1070	4.7	1.28	0.16
8	M	61	ciliary body	1.2	7.17	500	2.20	0.60	0.08
9	M	73	conjunctiva	2.3	4.87	573	2.52	0.69	0.09
Mean +/−SD					5.9+/−1.8	522+/−233	2.29+/−1.03	0.63+/−0.28	0.08+/−0.03

**Figure 1:**
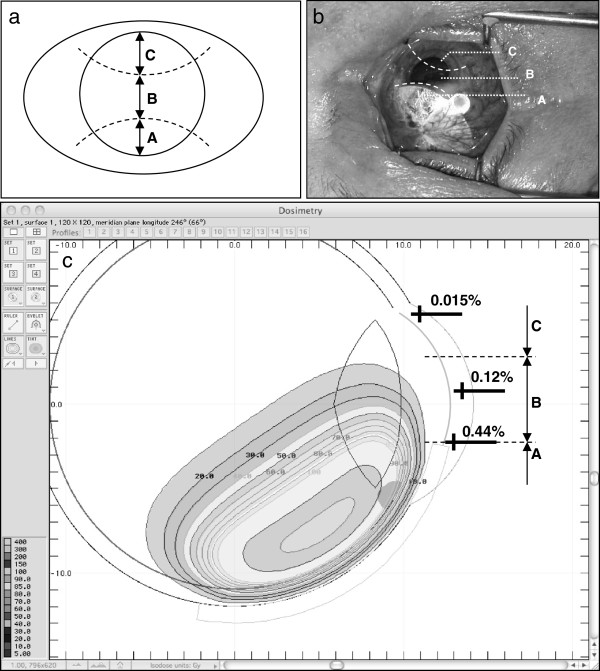
**Clinical situation and isodose distribution by Ru-106 brachytherapy.** (**a**) Schematic diagram of the corneal zones: applicator zone (A), central zone (B) and distal zone (C), each representing approximately one-third of the corneal vertical diameter. Note that vertical diameter of the cornea is approx. 10.5 mm, and the horizontal diameter 11.5 mm. (**b**) Photograph of a patient’s eye during brachytherapy: the Ru-106 plaque is located in the upper third of the cornea. (**c**) Diagram of isodose distribution: Simulation of CCB plaque with a dose of 522 Gy to the tumour base. The directly adjacent cornea receives 2.29 Gy (0.44% isodose), the central zone 0.63 Gy (0.12%) and the distal zone 0.08 Gy (0.015%) over the entire time course of plaque brachytherapy.

In order to simulate the radiation level in the cornea an isodose curve plot for the Ru-106 plaque was created using ‘Plaque Simulator’ Software (BEBIG GmbH, Berlin, Germany). The cornea was divided into three areas (each approximately one-third of the corneal diameter): (A) the applicator zone (the cornea directly beneath the plaque); (B) the central zone (equivalent to the central third of the cornea); and (C) the distal zone (the peripheral cornea distant from the applicator zone) (Figure 1a, b). The structures of the SNP are located in the cornea at a depth of 55–70 μm, and therefore the dose calculation was performed as for the corneal surface. The dose in the central axis of the cornea to the tumour base in the applicator zone differed between patients depending on melanoma size (see Table 1), ranging from 278 to 1070 Gy with a mean value of 522 Gy, a median dose of 520 Gy, and a mean dose rate of 5.9 Gy/h. The isodose plot shows that the applicator zone has about 100% of the tumour base dose (522 Gy), the corneal surface directly adjacent to the plaque 0.44% (2.29 Gy), the surface of the central third of the cornea a mean of 0.12% (0.63 Gy) and the distal zone 0.015% (0.08 Gy) (Figure 1c). The individual prescribed dose to the tumour base as well as the calculated doses in each corneal zone are presented in Table 1.

In-vivo confocal microscopy of SNP structures and assessments of corneal sensation were performed before irradiation, and at 3 days and 1, 4 and 7 months after therapeutic intervention.

In-vivo confocal microscopy with a Rostock Cornea Module (RCM) in combination with a Heidelberg Retina Tomograph II (Heidelberg Engineering, Heidelberg, Germany) equipped with a water contact objective (Zeiss, 63×/0.95 W, 670 nm, ∞/0, Jena, Germany) was performed as described elsewhere [20]. Image acquisition of the central axis of the cornea in the applicator, central and distal zones was performed in z-scan of automatic volume scan mode (30 images, volume depth 60 μm, constant interslice distance 2 μm, 384 × 384 pixels, 400 × 400 μm). Acquisition time for a single stack was 1.2 s. Confocal microscopy was performed in the region of interest, i.e. at the level of basal cells, SNP, Bowman’s membrane and anterior stroma at depths from 30 to 90 μm. At least three scans were performed in each zone. The total duration of microscopy was about 15 minutes.

**Figure 2:**
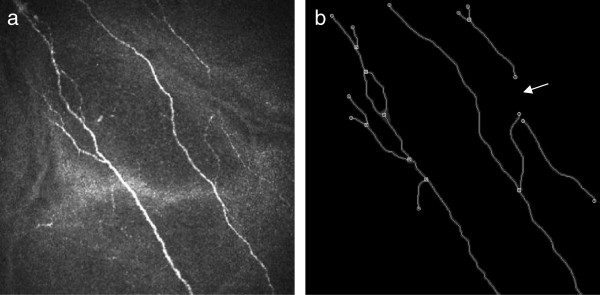
**Imaging and automated quantification of SNP.** (**a**) Representative in-vivo confocal image of the corneal SNP (distal zone, 4 months after plaque brachytherapy). (**b**) Results of automated image segmentation and analysis: total fibre length 1578 μm, nerve fibre density 9.863 mm/mm^2^, and single nerve fibre count (n) 19. Image size: 400 x 400 μm.

Automatic detection and quantification of SNP structures based on morphological and topological parameters was performed on the basis of an algorithm developed in-house. Image analysis was carried out in two stages. Firstly, the segmented images were analysed morphologically and after this step the fibres were skeletonised in order to obtain their one pixel wide medial axis. In a second stage the topological analysis of the medial axis network took place. Consequently the main parameters were split up into two main categories: (1) before skeletonisation (component pixels) and (2) after skeletonisation (number of nerve fibre components, skeleton pixels, single nerve fibres per component, total fibre length, average single fibre length, nerve fibre density, connectivity points, and number of branches) (Table 2). All measured variables can be used in isolation, combined or weighted for quantification of SNP networks. Representative images of the SNP as well as segmentation and analysis of the SNP are shown in Figure 2.

**Table 2:** Parameters used for automatic quantification of SNP structures

Component pixels	number of pixels identified as nerve fibres
Nerve fibre components	number of separate nerve fibre networks
Skeleton pixels	number of pixels representing the medial axes of nerve fibres
Single nerve fibres	number of all nerve segments between nerve branches and nerve ends
Total nerve fibre length/average single nerve fibre length	length of all nerve fibres in the image area/average length of single nerve fibres
Nerve fibre density	length of all fibres in respect to 1 mm^2^
Number of connectivity points	number of nerve fibres entering or leaving the image area to the outside

Corneal sensation was evaluated with a Cochet-Bonnet aesthesiometer (Luneau Ophthalmologie, Chartres Cedex, France; diameter of monofilament 12/100). The filament was applied along its full 60 mm length; if the response was negative the filament length was then reduced in 5 mm steps. Sensation was tested in each region of the cornea, and normal sensation was defined as between 50 and 60 mm.

## Results

**Figure 3:**
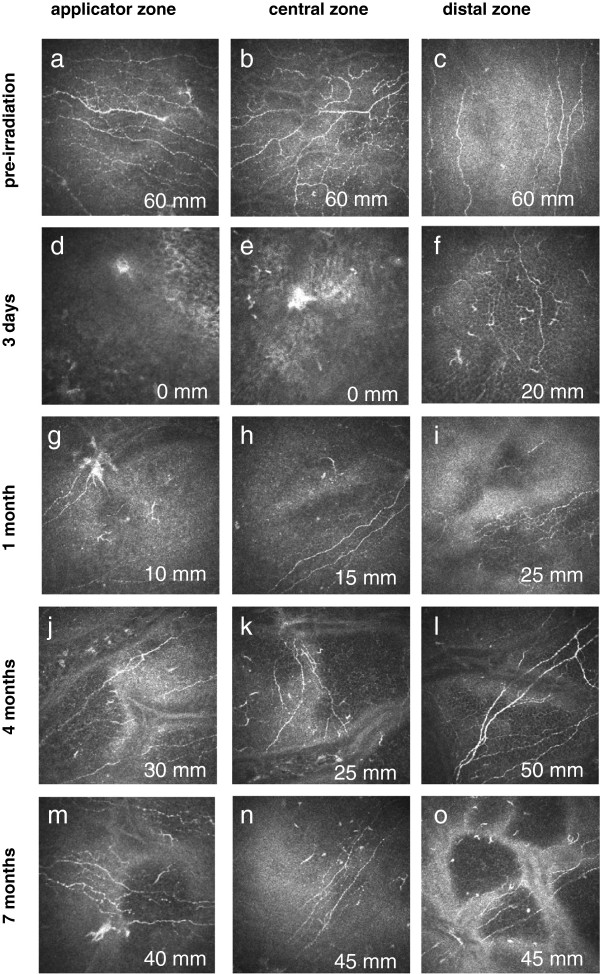
**Representative in-vivo confocal images of the corneal SNP of patients over time.** Image size: 400 × 400 μm. Each image also shows data on corneal sensation (in mm).

Before irradiation, the SNP displayed a typical pattern with hyperreflective fibres, regular tortuosity and nerve branching (Figure 3a-c). Three days after brachytherapy, no subbasal nerve fibres were visualised in the applicator zone or in the central zone (Figure 3d, e) and very few in the distal zone (Figure 3f). The earliest regenerating nerve fibres were detected at the one-month follow-up after therapeutic intervention: SNP structures were present in the whole cornea, although less branching and tortuosity were seen in the applicator zone and the central zone (Figure 3g, h). Four months postoperatively SNP structures had increased to almost one-third compared with the pre-irradiation status (Figure 3j, k). In qualitative terms SNP morphology had returned to initial status by seven months after plaque brachytherapy (Figure 3m, n).

Table 2 defines and Table 3 presents the quantitative data on micromorphological SNP variables before irradiation and during follow-up. In order to illustrate the most representative data as nerve fibre density (NFD, mm/mm^2^) and number of single nerve fibres (SNF, n, normalised to an area of 1 mm^2^) are reported in detail below.

**Table 3:** **Results of automated quantification of SNP variables before and after brachytherapy (all normalised to an area of 1 mm**^**2**^**)**

	**Corneal zone**	**Before brachytherapy**	**3 days after brachytherapy**	**1 month after brachytherapy**	**4 months after brachytherapy**	**7 months after brachytherapy**
Component pixels (n)	Applicator	45050.0 ± 23273.0	2031.3 ± 2637.8	11714.1 ± 12851.5	14118.8 ± 7941.5	38592.5 ± 25383.8
	Central	42589.6 ± 16506.1	1552.1 ± 2688.3	15663.8 ± 15369.3	14048.4 ± 7681.3	30116.7 ± 18365.5
	Distal	41421.3 ± 6598.9	11056.3 ± 6637.8	21918.8 ± 21252.3	30116.7 ± 24992.7	37814.6 ± 18680.4
Total number of nerve fibre components (n)	Applicator	50.0 ± 34.7	4.2 ± 3.6	23.4 ± 16.4	31.3 ± 22.8	53.8 ± 26.0
	Central	52.1 ± 25.5	2.1 ± 3.6	15.0 ± 13.0	12.5 ± 5.1	66.7 ± 36.1
	Distal	50.0 ± 40.3	25.0 ± 22.5	14.1 ± 16.4	22.9 ± 7.2	70.8 ± 18.0
Skeleton pixels (n)	Applicator	13715.6 ± 7004.1	622.9 ± 809.2	3456.3 ± 3774.2	4300.0 ± 2411.0	11622.5 ± 7566.4
	Central	12876.0 ± 5053.8	460.4 ± 797.5	4627.5 ± 4483.7	4217.2 ± 2308.7	9110.4 ± 5497.3
	Distal	12722.5 ± 1979.2	3347.9 ± 2056.2	6585.9 ± 6253.7	9077.1 ± 7387.1	11422.9 ± 5671.4
Single nerve fibres (n)	Applicator	240.6 ± 184.0	4.2 ± 3.6	37.5 ± 39.9	40.6 ± 25.8	188.8 ± 133.4
	Central	235.4 ± 167.5	6.3 ± 10.8	51.3 ± 68.4	96.9 ± 112.9	116.7 ± 85.1
	Distal	162.5 ± 84.7	47.9 ± 28.2	126.6 ± 127.5	175.0 ± 197.3	189.6 ± 108.7
Average single fibre length (μm)	Applicator	83.4 ± 34.3	166.4 ± 154.1	101.1 ± 52.4	147.3 ± 77.4	74.2 ± 13.5
	Central	74.4 ± 31.2	84.3 ± 0	151.9 ± 65.7	83.8 ± 46.1	89.2 ± 19.4
	Distal	100.2 ± 37.8	93.5 ± 60.7	61.1 ± 7.9	74.4 ± 22	68.8 ± 7.2
Nerve fibre density (mm/mm^2^)	Applicator	15.142 ± 7.93	0.693 ± 0.908	3.896 ± 4.315	4.713 ± 2.638	12.945 ± 8.586
	Central	14.207 ± 5.553	0.527 ± 0.913	5.322 ± 5.205	4.736 ± 2.538	10.031 ± 6.221
	Distal	13.827 ± 2.279	3.725 ± 2.302	7.471 ± 7.278	10.151 ± 8.627	12.643 ± 6.348
Connectivity points (n)	Applicator	14.2 ± 10.4	0 ± 0	1.3 ± 2.5	5.6 ± 6.6	13 ± 11.5
	Central	14.2 ± 6.5	0 ± 0	2.0 ± 3.3	1.3 ± 2.5	13.3 ± 10.4
	Distal	13.0 ± 9.6	1.7 ± 1.4	5.6 ± 6.6	8.3 ± 8.8	12.5 ± 9.0
Branches (n)	Applicator	104.2 ± 91.8	0 ± 0	7.8 ± 15.6	4.7 ± 6.0	73.8 ± 64.1
	Central	101.0 ± 101.6	2.1 ± 3.6	18.8 ± 35.1	45.3 ± 59.2	25.0 ± 33.1
	Distal	61.3 ± 43.6	12.5 ± 16.5	62.5 ± 61.0	87.5 ± 120.5	58.3 ± 54.6

**Figure 4:**
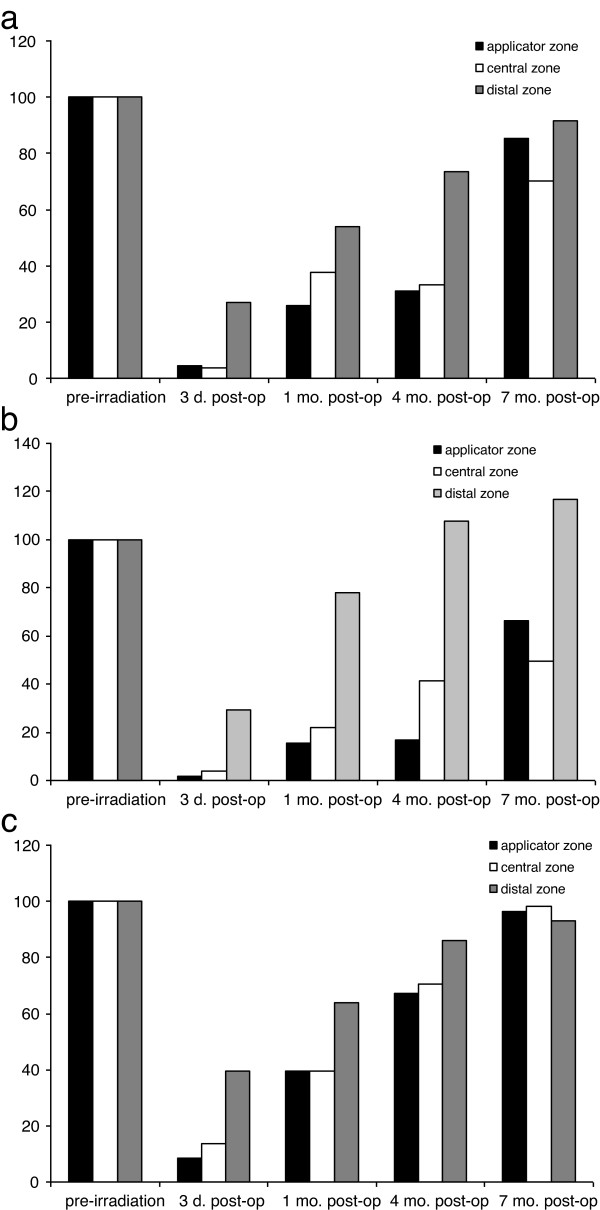
**Automated quantification of SNP structures and corneal sensation over time (percentage changes compared with pre-irradiation level): **(**a**) Automated quantification of nerve fibre density. (**b**) Automated quantification of single nerve fibres. (**c**) Corneal sensation.

NFD (Figure 4a): the applicator zone was characterised by a decrease in NFD to 5% of pre-irradiation density at 3 days after therapeutic intervention, followed by post-therapeutic regeneration to 26%, 31% and 85% at 1, 4 and 7 months respectively. A similar pattern was found for the central zone: a decrease to 4% at 3 days with subsequent post-therapeutic increases in NFD to 37%, 33% and 71% at 1, 4 and 7 months respectively. Interestingly, the distal zone also showed a reduction in NFD to 27% at 3 days after therapeutic intervention, followed by regeneration to 54%, 73% and 91% at 1, 4 and 7 months respectively.

SNF (Figure 4b): the applicator zone showed a decrease in SNF to 2% at 3 days after therapeutic intervention, followed by a slow recovery to 16%, 17% and 78% at 1, 4 and 7 months respectively. The central zone was characterised by an initial reduction in SNF to 3% at 3 days, with subsequent rises to 22%, 41% and 50% at 1, 4 and 7 months respectively. The distal zone revealed a decrease to 29% at 3 days followed by an increase to 78% at 1 month. At 4 and 7 months after therapeutic intervention there was regeneration of SNF to 108% and 117% respectively.

Corneal sensation (Figure 4c): pre-irradiation corneal sensation was 58 ± 4 mm in all zones. After therapeutic intervention, corneal sensation in the applicator zone was markedly reduced, being 5 ± 6 mm at 3 days, and increasing to 23 ± 15 mm at 1 month, 39 ± 17 mm at 4 months, and 56 ± 4 mm at 7 months. The pattern of corneal sensation findings at the corresponding time points was 8 ± 6 mm, 23 ± 17 mm, 41 ± 19 mm and 57 ± 5 mm in the central zone; and 23 ± 12 mm, 37 ± 17 mm, 50 ± 18 mm and 54 ± 17 mm in the distal zone.

## Discussion

Cancer-related neuropathy is a serious complication resulting from nerve damage caused by a malignancy or its treatment [21]. A number of authors have evaluated different forms of neuropathy, most commonly describing nociceptive and neuropathic pain in radiation-induced neuropathy, resulting in radiculopathies, back pain, headaches, paraesthesia, etc. [22,23]. Previous experimental animal studies have disclosed radiation-induced changes in the rabbit sciatic nerve, e.g. vacuolation, degeneration and necrosis of axons with myelin fragmentation [24]. This and most other clinical and experimental studies have focused on radiation effects on myelinated nerves and nerve fibres. However, the mechanisms underlying degeneration and regeneration of myelinated and unmyelinated nerves may be different.

To our knowledge no clinical study has yet investigated the neuropathy affecting unmyelinated corneal nerves after brachytherapy. Previous studies on radiation damage in the eye have addressed neuropathy of the optic nerve as well radiation retinopathy, maculopathy, cataracts, vitreous haemorrhage or secondary glaucoma after plaque therapy [7] and external beam radiotherapy [25].

Several studies have described degeneration of SNP structures in diabetes mellitus [16,26,27] or Fabry disease [17,28]. Regeneration of SNP has also been assessed after refractive surgery [29] and corneal grafting [13], procedures that were also associated with peripheral neuropathies. Our study demonstrates that even low radiation doses such as those received by the central zone lead to rapid but reversible degeneration of the SNP in the cornea.

Degeneration of nerve structures, characterised by complete absence of subbasal nerve fibres, was evident in the applicator zone and central zone directly after plaque brachytherapy. The first thin regenerating fibres of SNP were seen at the one-month follow-up, and by 4 months after therapeutic intervention SNP structures had increased to about one-third of pre-irradiation status (based on nerve fibre density and number of nerve fibres). While almost complete SNP regeneration was noted at 7 months after the intervention, the micromorphology of the nerve fibres in all zones irrespective of the local radiation dose was still quantitatively altered compared to pre-irradiation findings (Figure 4b, c).

Post-irradiation damage to the SNP, together with its regeneration, showed a direct correlation with the changes in corneal sensation recorded during follow-up in the present study. Sensation in the applicator zone was reduced to below 10% of pre-irradiation values at 3 days after the intervention, before increasing again to about 67% at 4 months and 96% at 7 months (Figure 4c). Post-irradiation corneal sensation in the distal zone was about 40% at 3 days, 86% at 4 months and 93% at 7 months. These findings strongly suggest that the local biological radiation damage is not related to the local dose but rather reflects a particular form of bystander radiation damage. The entire SNP network responds as one biological structure with little difference between high-dose areas and low-dose areas.

The radiation doses in the central cornea as well as in the distal zone are obviously very low. Similar doses are encountered in external beam radiotherapy of head and neck cancers, and yet no impairment of corneal sensation has ever been reported. Therefore we presume that the decrease in SNP density might be induced as a result of radiation damage to the nerve fibre network directly in the applicator zone. Since the central part of the SNP originates from collaterals of the SNP in the periphery as well as from the sub-Bowman’s nerves of anterior stroma [30], we postulate that the damage to nerves in the central and distal zones is due to the disruption of the complex collateral 3-dimensional organisation of SNP.

One limitation of our study is the 1 μm lateral resolution of in-vivo confocal microscopy. This means that nerve structures below 1 μm were not detected, thus accounting for some discrepancy of findings. A possible way forward might be 3-dimensional reconstruction of the image with resultant extraction of SNP structures and subsequent automated analysis [31]. Moreover, in follow-up investigations of corneal surfaces which receive vastly different radiation doses, microscopy of a larger area would be useful. New developments of technology permit mapping of the cornea with a maximum image size of 3072 × 3072 pixels (3.2 × 3.2 mm) [18].

Several pathogenic pathways and manifestations of corneal neuropathy can be distinguished: (i) mechanical damage to corneal nerves after corneal grafting; (ii) mechanical or laser-induced flap preparation during laser in-situ keratomileusis (LASIK); (iii) mechanical abrasion and laser treatment procedures during photorefractive keratectomy (PRK); and (iv) UVA light and riboflavin exposure.

Neuropathy after corneal grafting was reported in histochemical studies by Tervo et al.: regeneration of SNP and stromal nerves was still incomplete as late as 36 months after surgery [32]. Ruben et al. showed that about one-third of operated corneas were still ‘clinically anaesthetic” (≤ 20 mm) 6 years postoperatively and another one-third showed reduced corneal sensation [33]. Furthermore, the regenerated nerves were thinner and had abnormally curved branches; also the structures with a whorl-like pattern were absent.

SNP structures after LASIK display the same micromorphological pattern as after corneal grafting. While the earliest regenerating nerves were detected 1 month postoperatively, morphologically complete regeneration was still not evident after 2 years. Subbasal nerve fibre density after LASIK has been reported to be reduced by 51%, 35% and 34% at 1, 2 and 3 years respectively [34]. Interestingly, following PRK (i.e. direct damage to the SNP in the central 6 mm of the cornea and no mechanical flap with intact hinge zone) subbasal nerve fibre density was reduced by 59% at 1 year compared with preoperative status. By 2 years subbasal nerve fibre density was virtually unchanged from preoperative values, and remained constant over the next 3 years of follow-up [34]. Our own group has demonstrated that corneal sensation returns to 90% of normal values by about 12 months postoperatively. However, other studies have described delayed recovery of corneal sensation over periods ranging from 3 weeks to 9 months [11].

Another example of SNP damage is that which results from collagen cross-linking (exposure to riboflavin and UVA light): the mechanism responsible for corneal neuropathy in that setting remains unclear. Nerve branches are reportedly absent immediately postoperatively, while the first isolated nerves have been noted from 3 months onwards [35] and have ‘regenerated’ by 6 months after cross-linking [36].

Taken together, different mechanisms of nerve damage after therapeutic intervention lead to a decrease in nerve fibre density and a resultant reduction in corneal sensation. Regenerated nerves are characterised by fewer branches and greater tortuosity. The recovery of corneal sensation correlates not only with the number of nerve fibres but with the presence and number of collaterals. Our study demonstrates that radiation-induced neuropathy of corneal nerves develops quickly after plaque brachytherapy and is partly reversible within 7 months. The SNP network responds not locally, but as an interactive biological system, both with regard to damage expansion into low-dose areas and regeneration into high-dose areas. Further studies will be needed to investigate the dependence of degeneration and regeneration on local dose and dose inhomogeneity. As a positive conclusion it can be stated that the clinical impact of radiation-induced SNP damage is moderate.

## Competing interests

The authors declare that they have no competing interests.

## Authors’ contributions

Conceived and designed the experiments: AZ, GH, RG and OS. Performed the experiments: AZ, SP, HG and RG. Analyzed the data: AZ, KW, SP, OS, AW, GH and RG. Wrote the paper: AZ, KW, SP, OS, AW, GH and RG. All authors read and approved the final manuscript.
